# Enzyme-Functionalized Piezoresistive Hydrogel Biosensors for the Detection of Urea

**DOI:** 10.3390/s19132858

**Published:** 2019-06-27

**Authors:** Jan Erfkamp, Margarita Guenther, Gerald Gerlach

**Affiliations:** Solid-State Electronics Laboratory, Technische Universität Dresden, 01069 Dresden, Germany

**Keywords:** stimuli-responsive hydrogel, urea, urease, biosensor, pH value, alkaline pH conditions, urea sensor, piezoresistive pressure sensor, hydrogel-based sensor

## Abstract

Urea is used in a wide variety of industrial applications such as the production of fertilizers. Furthermore, urea as a metabolic product is an important indicator in biomedical diagnostics. For these applications, reliable urea sensors are essential. In this work, we present a novel hydrogel-based biosensor for the detection of urea. The hydrolysis of urea by the enzyme urease leads to an alkaline pH change, which is detected with a pH-sensitive poly(acrylic acid-*co*-dimethylaminoethyl methacrylate) hydrogel. For this purpose, the enzyme is physically entrapped during polymerization. This enzyme-hydrogel system shows a large sensitivity in the range from 1 mmol/L up to 20 mmol/L urea with a high long-term stability over at least eight weeks. Furthermore, this urea-sensitive hydrogel is highly selective to urea in comparison to similar species like thiourea or *N*-methylurea. For sensory applications, the swelling pressure of this hydrogel system is transformed via a piezoresistive pressure sensor into a measurable output voltage. In this way, the basic principle of hydrogel-based piezoresistive urea biosensors was demonstrated.

## 1. Introduction

Enzymes are a powerful tool for the development of novel biosensors with high sensitivity and substrate specificity. Well-known examples for enzymatic biosensors are commercially available glucose sensors for the monitoring of the blood-sugar-level [1] or lactate sensors for biomedical diagnostics [2]. Furthermore, enzymatic biosensors are used for monitoring of biotechnological processes or in food industry [3]. It is therefore not surprising that biosensors are one of the largest growth markets in sensor technology. Biosensors worth over US$ 13 billion were sold in 2013 and, according to prognoses, the sales volume should be raised continuously over the following years [4]. 

The biological signal due to the enzymatic reaction, however, must be transformed via a transducer to generate a measurable output signal. One example is the usage of hydrogel-based piezoresistive pressure sensors. Smart hydrogels swell and shrink in dependence on different stimuli [5]. The resulting swelling pressure of the gel deforms the bending plate of the pressure sensor. The deformation of the piezoresistors within the bending plate leads to a change in resistance that will be transformed via a Wheatstone bridge circuit into a measurable output voltage [6]. Based on this principle, hydrogel-based sensors for the detection of the pH value [7], temperature [8] or organic solvents like ethanol [9,10] could be realized. 

For the development of novel hydrogel-based biosensors, stimuli-responsive hydrogels can be easily functionalized with enzymes. In the case of glucose oxidase, the enzymatically catalyzed hydrolysis of the substrate leads to an acidic pH change [11], which can be detected with pH-sensitive hydrogel-based pressure sensors. Based on pH-sensitive poly(hydroxypropyl methacrylate-*co*-dimethylaminoethyl methacrylate) hydrogels and the enzyme glucose oxidase, Schmidt et al. could successfully realize hydrogel-based glucose sensors with a sensitivity in the low millimolar range [12].

**Figure 1:**
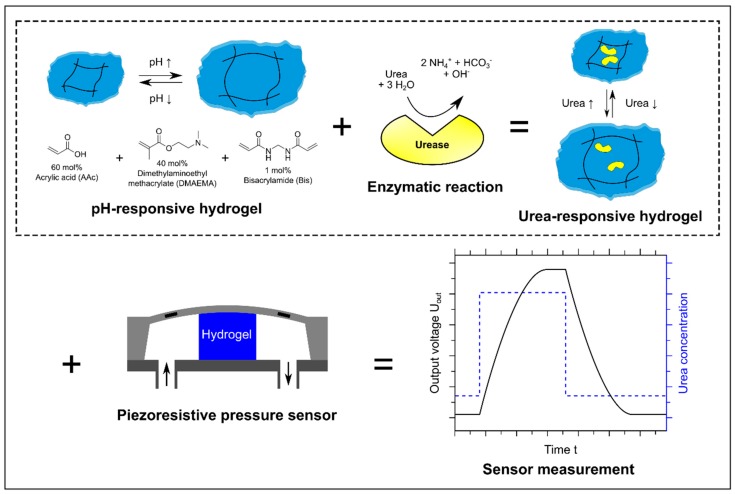
Measuring principle of a hydrogel-based urea biosensor: pH-responsive hydrogels based on acrylic acid (60 mol%) and dimethylaminoethyl methacrylate (40 mol%) are functionalized with the enzyme urease. The enzymatic hydrolysis of urea leads to an alkaline pH change and the gel swells due to this pH change. The resulting swelling pressure is transformed into an output voltage via a piezoresistive transducer. The schematic illustration of the hydrogel-based pressure sensor was modified according to [9] and the enzymatic reaction and the hydrogel according to [21].

Urea is one of the most important organic compounds in the world. For instance, urea is used industrially as fertilizer for plants, as feed supplement for animals or for the production of cosmetics and pharmaceuticals [13]. Furthermore, the urea concentration is an important biomedical indicator for diseases in blood or urine [14]. Therefore, a reliable detection of the urea concentration is essential for these applications. For the usage of urea biosensors, the enzyme urease is often used. The enzymatic reaction leads to an alkaline pH change (Figure 1), which can be detected for example with a pH electrode [15,16] or an ion-sensitive field-effect transistor [17,18]. 

In this work, we used the enzyme urease to functionalize pH-responsive poly(acrylic acid-*co*-dimethylaminoethyl methacrylate) hydrogels [19,20]. The major goal of this work was to study how hydrogels can be functionalized with enzymes to design new kinds of stimuli-responsive hydrogels for sensing applications. The catalyzed hydrolysis of urea by the immobilized enzyme leads to a swelling of the gels in dependence on the urea concentration. These urea-responsive hydrogels showed a high sensitivity and good long-term stability over several weeks. Furthermore, we demonstrated the working principle of urea-functionalized piezoresistive hydrogel biosensors and discussed their sensor properties for possible applications. The presented sensing concept is summarized in Figure 1. 

## 2. Materials and Methods

### 2.1. Synthesis of Urea-Sensitive Hydrogels Based on Poly(Acrylic acid-co-Dimethylaminoethyl methacrylate) and the Functionalized Enzyme Urease

At first, the pH-sensitive monomers acrylic acid (60 mol% AAc, 576 µmol, 39.5 µL, Sigma Aldrich, St. Louis, MO, USA) and dimethylaminoethyl methacrylate (40 mol% DMAEMA, 384 µmol, 64.7 µL, Sigma Aldrich) were combined with the crosslinker bisacrylamide (1 mol% Bis, 9.6 µmol, 96.6 µL of the 0.0994 mol/L stock solution in PBS buffer, Carl Roth, Karlsruhe, Germany) and the solution was degassed with nitrogen for 5 min. The total monomer concentration was 1.6 mol/L (=100 mol%) [19,20]. To prepare the PBS buffer, a PBS tablet (Sigma Aldrich) was dissolved in 200 mL distilled water. Separately from the monomer solution, the enzyme urease (generally with a concentration of 1 kU/mL for a synthesis approach, urease type III from jack beans, Sigma Aldrich) was dissolved in PBS buffer wherein the buffer has already been degassed with nitrogen for 5 min prior to the addition of the enzyme in order to reduce the mechanical stress for the enzyme. Afterwards, the monomer solution and the enzyme containing buffer were combined carefully and the polymerization was initiated by adding ammonium peroxodisulfate (1 mol% APS, 9.6 µmol, 2.2 mg, Sigma Aldrich) and *N,N,N’,N’*-tetramethylethylenediamine (1 mol% TEMED, 9.6 µmol, 1.5 µL, Carl Roth). The pregel solution with a total volume of 600 µL was filled in a vial shell (1 mL, VWR, Radnor, PA, USA) and was put for 3 h in an oven at 35 °C to ensure a complete degradation of the thermal initiator and, therefore, a fast polymerization of the gel. To reduce the thermic stress for the enzyme, the polymerization was completed at room temperature after 21 h. Afterwards, the gels were cut in small pieces (thickness of about 5 mm) and were washed for 3−4 days with a PBS buffer solution to remove unreacted monomers. To verify the experimental results, similar hydrogels without the enzyme urease were also polymerized according to the same synthesis protocol. From each synthesis approach, two hydrogel pieces were generated. At least five (e.g., for the swelling kinetics) up to twelve (e.g., for the long-term stability) of these hydrogel pieces were used for the swelling experiments. All further experiments were done at room temperature. 

### 2.2. Swelling Studies in Dependence on the Urea Concentration 

After synthesis and washing of the gels with and without the functionalized enzyme urease, the hydrogel mass *m_0_* was determined using a balance (Entris 224-1S, Sartorius, Goettingen, Germany) and the pH value of the solution was measured using a pH meter (FiveEasy Plus, Mettler-Toledo, Gießen, Germany). Urea (Sigma Aldrich) was dissolved in PBS buffer and each gel was incubated in 3 mL of the urea solution for 24 h. After the determination of the hydrogel mass *m* and the pH value of the surrounding solution, the solution was changed to the next higher urea concentration. This procedure was repeated several times. In total, the hydrogel sensitivity with regard to urea was tested in a range from 1 mmol/L up to 100 mmol/L urea. Based on the measured hydrogel masses, the swelling degree *S* was calculated according to Equation (1):

*S = (m − m_0_) / m_0_* × 100% (1)

### 2.3. Swelling Kinetics of Urea-Sensitive Hydrogels

For the measurement of the swelling kinetics, the initial mass *m_0_* of the hydrogels with and without the enzyme urease was determined in PBS buffer without urea. Afterwards, the buffer of each hydrogel sample vial was replaced to 3 mL of a PBS buffer containing 20 mmol/L urea. Subsequently, the hydrogel mass *m* was measured at defined times until the calculated swelling degree *S* according to Equation (1) remains constant. After the swelling kinetics, also the deswelling kinetics was tested. The surrounding buffer of the swollen hydrogels was changed to a PBS buffer without urea (3 mL for each hydrogel sample vial) and the hydrogel mass *m* was measured at defined times until swelling degree *S* remains constant. To ensure a complete pH change over time, the PBS buffer was changed after 1 h for the first time and then after every additional measuring point. 

### 2.4. Repeatability and Long-Term Stability of Urea-Sensitive Hydrogels

The repeatability of the hydrogel swelling was tested over eight weeks. Thus, a statement about the long-term stability can be concluded for that period of time. After the determination of the hydrogel mass *m_0_* and the pH value in PBS buffer without urea, the surrounding buffer of the gels was replaced by 3 mL of a PBS solution containing 20 mmol/L urea for each gel sample. After 24 h, the hydrogel mass *m* and the pH value after swelling were determined. In the next step, the buffer solutions of each hydrogel sample were changed to a PBS buffer without urea for in total six days. During this procedure, the PBS buffer solutions were changed three times in the first 24 h and afterwards almost daily to ensure a complete pH change. The hydrogel mass *m* and the pH value were determined, and the buffer of the samples was changed to a PBS buffer containing 20 mmol/L urea. These cyclic changes of the buffer solutions were performed several times for in total eight weeks. The resulting swelling degree *S* of the gels was calculated according Equation (1). During this experiment, the hydrogels were stored at room temperature. 

### 2.5. Selectivity of Urea-Sensitive Hydrogels to Similar Species

The selectivity of the enzyme-functionalized hydrogel system was tested with thiourea (Gruessing, Filsum, Germany), *N*-methylurea (Sigma Aldrich) and *N,N,N’,N’*-tetramethylurea (Merck, Darmstadt, Germany), which are chemically similar species to urea. Therefore, PBS buffer solutions with 20 mmol/L of urea, thiourea, *N*-methylurea and *N,N,N’,N’*-tetramethylurea were produced. Now the swelling kinetics of the gels was recorded in dependence on the substrate solutions as described in Section 2.3. 

### 2.6. Effects of Different Amounts of Enzyme on the Sensitivity and Swelling Kinetics of Urea-Sensitive Hydrogels 

During synthesis (see Section 2.1), the amount of enzyme was varied between 0.1 and 2 kU/mL of the enzyme urease per synthesis approach. The swelling experiments in dependence on the urea concentration and the swelling kinetics were performed as described in Section 2.2 and Section 2.3, respectively.

### 2.7. Hydrogel-Based Piezoresistive Urea Biosensors

For the application of these gels in a piezoresistive pressure sensor, enzyme-functionalized hydrogels with defined shape and thickness were synthesized. During polymerization, the pregel solution was filled in a cavity of a Teflon spacer with a thickness of 500 µm. After polymerization and washing the gels for three days, circular hydrogel pieces with a diameter of 1.5 mm were punched out of the gel. The gel pieces were fixed with cyanoacrylate on a circuit board. The pressure sensor chip (TDK Electronics, previously EPCOS, Munich, Germany, C41-Series, 5.0 mm × 5.0 mm × 0.4 mm) was put on top of the gel piece and also fixed with cyanoacrylate on the circuit board. After wire bonding to connect the sensor chip with the circuit board, connector cables were soldered to the readout unit (Fluke 45, Glottertal, Germany). By using cyanoacrylate and two-component epoxy resin, an inlet and outlet hose for the measuring solutions were fixed on the resulting urea biosensor [9,10,20]. 

The hydrogel biosensor was washed over night in PBS buffer. Afterwards, the sensor was conditioned three times by cyclic changes of a 20 mmol/L urea solution in PBS buffer and a PBS solution without urea for in total three days. At first, the repeatability of the sensor measurement was shown. Therefore, a 20 mmol/L urea solution in PBS buffer was pumped though the sensor for 4 h. Afterwards, the buffer was changed to a PBS solution without urea for 20 h. This procedure was performed in total three times. Subsequently, the sensor was washed for 24 h with PBS buffer. In the next step, the sensitivity range of the biosensor was investigated. PBS buffer solution containing urea in a range from 1 mmol/L to 20 mmol/L were prepared. Starting with the lowest urea concentration, the buffer was changed to the next higher urea concentration after 4 h.

For the sensor measurements, a peristaltic pump (Reglo Digital MC-2/6, Ismatec as part of Cole-Parmer, Wertheim, Germany) was used. The flow rate was constant (0.2 mL/min) over time. The supplied voltage of the sensor was 5 V (DIGI 35, Voltcraft as part of Conrad Electronic, Hirschau, Germany). The measuring signal of the sensor was recorded every 10 s. All sensor measurements were performed at room temperature.

## 3. Results and Discussion

### 3.1. Verification of the Enzyme Immobilization 

**Figure 2:**
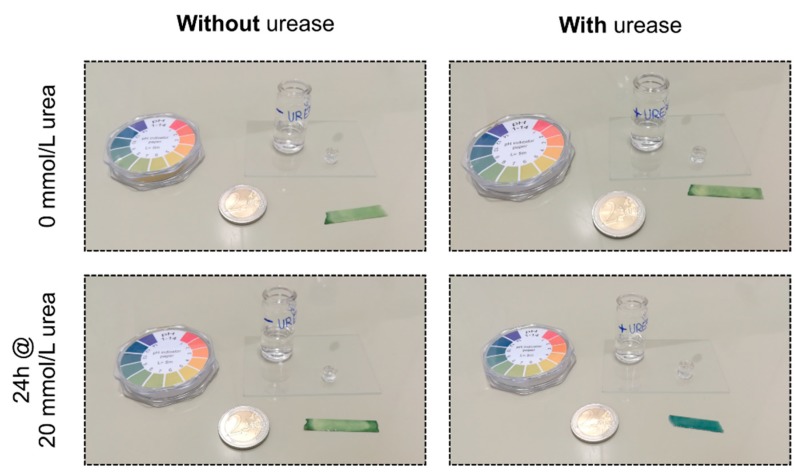
Enzymatic reaction after physical entrapment of the enzyme into the gel via paper-based pH indicators. Due to the enzymatic reactions, the neutral solution (green pH indicator) turns to a measurable alkaline pH change (blue pH indicator).

The enzyme was added to the monomer solution during polymerization. This physical entrapment method of the enzyme into the gel is often used in literature. However, free radicals during the polymerization could damage the enzyme and this could lead to a loss of the enzyme activity [22]. Therefore, the enzymatic activity was tested after polymerization. After synthesis and washing of an enzyme-functionalized hydrogel and a hydrogel without enzyme, the pH value was tested with a paper-based universal indicator in a PBS solution without urea (Figure 2). Both pH indicators turned green, meaning that the pH value was neutral. Afterwards, the buffer solutions of the samples were changed to a PBS buffer with 20 mmol/L urea and the pH value was checked after 24 h. For the hydrogel sample without enzyme, no change in pH value could be observed. For the enzyme-functionalized hydrogel sample, however, the paper-based pH indicators turned blue. This significant alkaline pH change resulted from the enzymatic reaction of the immobilized urease (Figure 1). As a result, the immobilization of the enzyme into the gel was successfully realized without losing any significant enzyme activity during synthesis.

### 3.2. Swelling Studies in Dependence on the Urea Concentration 

**Figure 3:**
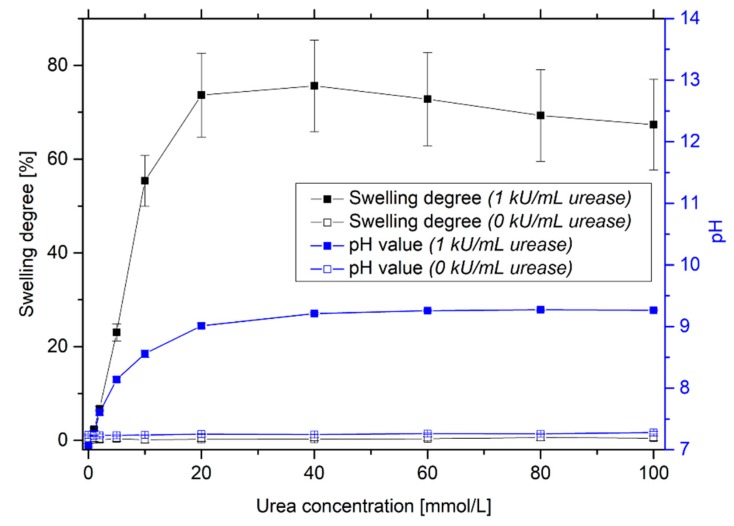
Swelling behavior of enzyme-functionalized and non-functionalized poly(AAc-*co*-DMAEMA) hydrogels and resulting pH value for different urea concentrations.

For the determination of the urea-sensitive measuring range, the hydrogels were incubated in PBS solutions with different urea concentrations (Figure 3). With an increase in urea concentration, the pH value of the surrounding solution increases due to the enzymatic reaction. This leads to a swelling of the used pH-sensitive hydrogel system, which was already shown in previous studies [20]. The system is highly sensitive up to 20 mmol/L urea. The detection limit is around 1–2 mmol/L. The measurement uncertainty is quite large. During polymerization, the enzyme seems to be inhomogeneously distributed in the gel. As a result, the swelling behavior of the gels can decisively vary from each other. Furthermore, local differences in the composition of the copolymer during the polymerization could lead to different sensitivities. As shown in Section 3.1, only urease-functionalized hydrogels are sensitive to urea. For non-functionalized hydrogels, no significant changes in pH value of the solution and in swelling degree of the hydrogel could be observed with increasing the urea concentration. 

### 3.3. Swelling Kinetics of Urea-Sensitive Hydrogels

**Figure 4:**
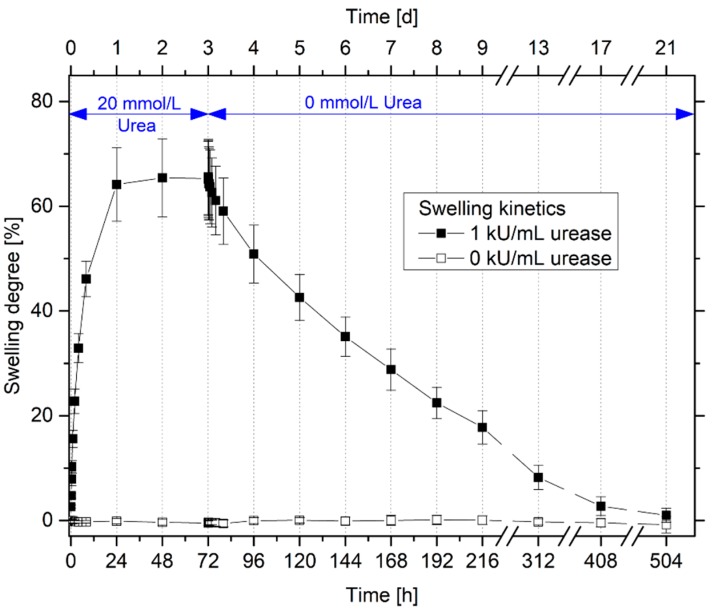
Swelling and deswelling kinetics of enzyme-functionalized and non-functionalized poly(AAc-*co*-DMAEMA) hydrogels with 20 mmol/L urea and without urea, respectively.

With the help of the swelling kinetics, important sensor properties such as the swelling behavior during the swelling and shrinking processes and the response times of the gels can be estimated. Therefore, the swelling and deswelling kinetics were characterized in detail (Figure 4).

The urease-functionalized hydrogels are completely swollen after 24 h in a 20 mmol/L urea solution in PBS buffer. As shown in Section 3.1 and Section 3.2 only urease-functionalized hydrogels swell in the urea solution due to the catalyzed enzymatic reaction. The gels require significantly more time for the shrinking than for the swelling process. The enzyme-functionalized gels are fully shrunken after in total two weeks. A similar asymmetric swelling behavior was observed for different kinds of hydrogels systems [20,23,24]. In several cases, this swelling behavior will be explained with the so-called “skin effect”. The stimulus in the surrounding solution always diffuses from the outer polymer-solvent interface into the center of the hydrogels. During the swelling process, the outer polymer surface of the hydrogel swells first and, therefore, the solution with the stimulus can be easily diffuse into the swollen polymer network. During the shrinking process, also the polymer surface of the gels shrinks at first. As a result, a dense polymer layer skin will be formed at the surface of the gel. This shrinking barrier hinders the diffusion of the stimulus and leads to a very slow deswelling process of the gel [25,26,27]. Furthermore, strong electrostatic interactions of the charged degradation products with the polyelectrolyte hydrogel could also hinder the diffusion of the mobile ions out of the gel [20].

For sensing applications, the response times are still too long. However, the size of the hydrogels substantially influences the swelling kinetics. For hydrogel-based piezoresistive sensors, much smaller hydrogels will be used compared to the free swelling experiments (gel disc thickness in free swelling experiments: ca. 5 mm and in the sensor application: 0.5 mm). As a result, the diffusion path will be reduced, and the stimulus can diffuse much faster into the gel. As an example, the time constant for spherical gels during the swelling process depends squarely on the radius of the gel [28]. This should significantly improve the response times of the hydrogel sensor [9,20].

### 3.4. Repeatability and Long-Term Stability of Urea-Sensitive Hydrogels

**Figure 5:**
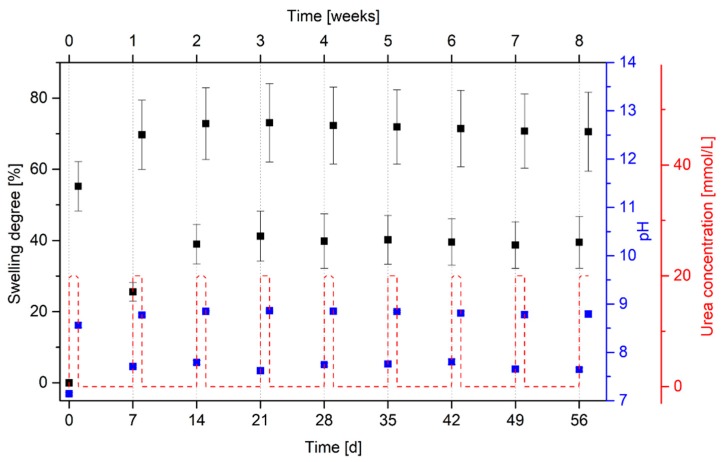
Long-term stability of urease-functionalized poly(AAc-*co*-DMAEMA) hydrogels during cycling between two alternating urea concentrations (20 mmol/L and 0 mmol/L) in PBS buffer. The resulting pH change in the surrounding solutions due to the enzymatic reaction was detected at the same time. Black squares describe the swelling degree of the gels, blue squares characterize the pH value of the surrounding solutions and the red line represents the urea concentration during this swelling experiment.

For a reliable sensor measurement over time, the long-term stability is one of the most decisive factors. Especially for enzyme-based sensors, however, the long-term stability is often problematic. The lifetime of enzymes are inherently limited. Enzymes can be deactivated over time by different chemicals or physical conditions in the sample [29]. Furthermore, the enzyme leakage out of the gel leads often to a loss of enzymatic activity over time [22,30]. For this reason, the long-term stability of the enzyme-functionalized hydrogels was tested for in total eight weeks (Figure 5). 

After eight weeks, no significant loss of enzymatic activity was detected. The hydrogels swell and deswell repeatably in dependence on the urea concentration due to the enzymatic reaction and the resulting pH change. As shown in the swelling kinetics in Section 3.3, the hydrogel systems needed two weeks for a complete shrinking process. As the gel shrinking was already measured after six days, it is not surprising that the initial mass of the gels was not reached. Based on these results, it can be assumed that the hydrogel sensor should be stable for eight weeks and more.

For a long-term stable immobilization of enzymes in a hydrogel via physical entrapment, the mesh size of the gel and the size of the enzyme is very important. Typically, the mesh size of a hydrogel is in the order of a few nanometers [31,32,33]. However, the mesh size depends strongly on the monomer composition, the crosslinker content, the stimuli and, thus, the swelling degree of the gel [34]. The enzyme urease is a comparatively large enzyme with a molecular weight of 551 kDa (1 Da = 1 g/mol [35]) and a hydrodynamic radius of about 7 nm [36]. For comparison, the enzyme glucose oxidase, which is typically used for the detection of the blood sugar level, has a significantly smaller molecular weight (147 kDa) and a smaller hydrodynamic radius (4.15 nm) [37]. The larger the enzyme and the smaller the mesh size of the gel are, the more difficult it is for the enzyme to diffuse out of the gel. This could be an explanation for the good long-term stability of these enzyme-functionalized hydrogels. Furthermore, the charged groups of the enzyme can be interacted with the charged functional groups of the hydrogel. These electrostatic forces could also hinder the enzyme to diffuse out of the gel.

In comparison to other enzyme-based urea sensors, this sensor concept shows a good long-term stability. Eggenstein et al. introduced a potentiometric urea biosensor based on an ammonia-sensitive electrode. The enzyme urease was encapsulated in a poly(carbamoylsulfonate) hydrogel. However, the operational stability of this sensor concept was limited to only four days at 4 °C [38]. Lee et al. developed a conductometric urea biosensor based on an interdigitated array electrode. The enzyme was immobilized in a sol-gel process by using tetramethoxysilane. After only three weeks, the biosensor retained only little more than 50% of its initial enzymatic activity at 4 °C [39]. Das and Sarkar modified an electrode with a conducting polymer hydrogel membrane based on poly(vinyl alcohol) and poly(acrylamide). Over the hydrogel membrane, the enzyme was covalently immobilized using glutaraldehyde. After two months storage at 4 °C, 94% of the original activity retained [40]. Singh et al. summarized further examples for urea biosensor concepts and their sensor characteristics [41]. All in all, the long-term stability of enzymes depends strongly on the immobilization strategy, the amount of enzyme during the immobilization and the storage conditions like temperature or buffer composition. 

An additional chemical covalent immobilization, e.g., with 1-ethyl-3-(3-dimethylaminopropyl) carbodiimide (EDC) and *N*-hydroxysuccinimide (NHS) [42], could also further increase the long-term stability of the tested enzyme-functionalized hydrogels in this work. However, this undirected immobilization, e.g., in the active center of the enzyme, can lead to a significant loss of activity during the immobilization [22]. To avoid this and because of the already existing good long-term stability of the characterized gels, additional immobilization steps were waived. 

In addition to the long-term stability of these enzyme-functionalized hydrogels, another effect was also observed: Hydrogels were often conditioned before usage in sensing applications [20,43,44]. The polymer network finds their optimal arrangement and too short polymer chains break due to the conditioning process. As a result, the measurement accuracy of hydrogel-based sensors will be significantly increased [28]. In all free-swelling experiments in this work, the hydrogels were not conditioned. For this reason, the swelling degree of the gels increased in the first two conditioning cycles while afterwards the swelling degree remained constant. To avoid this effect in the sensor measurements, the hydrogel-based piezoresistive urea biosensor was conditioned by cycling them three times before the actual sensor measurement (see Section 2.7).

### 3.5. Selectivity of Urea-Sensitive Hydrogels to Similar Species

**Figure 6:**
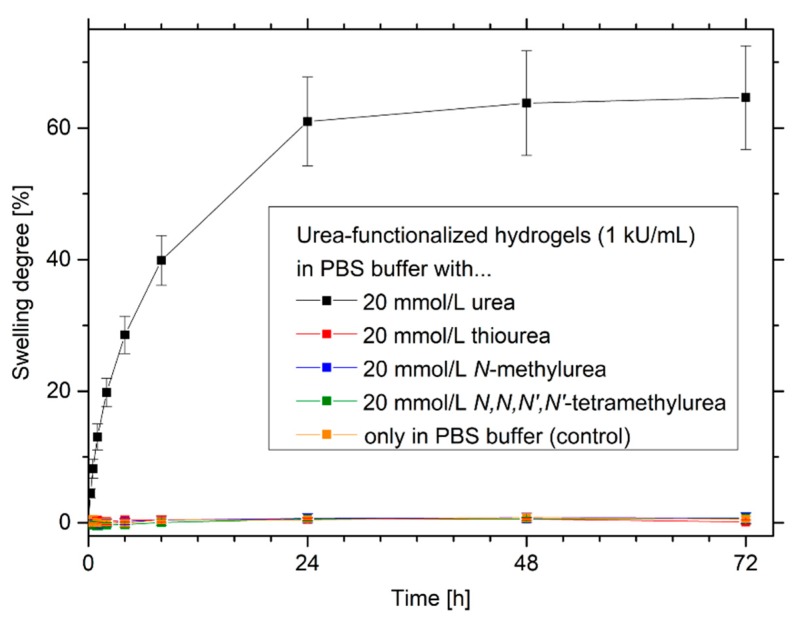
Substrate specificity of urease-functionalized poly(AAc-*co*-DMAEMA) hydrogels, tested via swelling kinetics in urea, thiourea, *N*-methylurea and *N,N,N’,N’*-tetramethylurea.

Enzymes are often not specific to similar species. For example, the enzyme alcohol dehydrogenase has a low specificity and cannot differentiate between different kinds of alcohols, especially primary alcohols. These cross-sensitivities can significantly influence the sensor measurements of alcohol biosensors [45,46,47]. For this reason, the selectivity of the urease-functionalized hydrogel system was tested with urea, thiourea, *N*-methylurea and *N,N,N’,N’*-tetramethylurea (Figure 6). 

Urease-functionalized hydrogels have a high selectivity to urea in comparison to the other similar substrates. Only in a urea-containing buffer solution, a significant swelling of the gels was observed. Similar substrate structures like thiourea, *N*-methylurea and *N,N,N’,N’*-tetramethylurea were not enzymatically degraded. Therefore, almost no change in pH value and no swelling of the pH-sensitive hydrogels were detected. The high substrate specificity of the enzyme urease is also reported in the literature. Urea analogues like thiourea, *N*-methylurea or hydroxyurea are well known as competitive inhibitors for the enzyme urease [48]. Due to their structural similarity to the substrate, competitive inhibitors can diffuse to the active center of the enzyme, however, these substances cannot be converted by the enzyme [49]. The inhibition of the enzyme by these structures should not be a problem in the application since the substrate urea is usually present in excess compared to the inhibitors and the substrate can therefore displace the competitive inhibitor.

### 3.6. Effects of Different Amounts of Enzyme on the Sensitivity and Swelling Kinetics of Urea-Sensitive Hydrogels 

**Figure 7:**
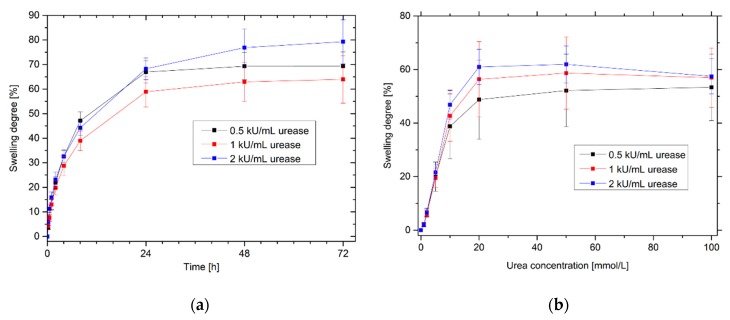
Influence of the amounts of enzyme for urea-sensitive hydrogels on the (**a**) swelling kinetics in a 20 mmol/L urea solution and (**b**) swelling behavior for different urea concentrations.

Enzymes for biosensory applications are comparatively expensive. In order to keep the costs for the production of biosensors as low as possible, the enzyme quantities should be as low as possible. However, minimization of the amounts of enzyme should not lead to deterioration of sensor properties such as sensitivity or response time. For this reason, the influence of the enzyme quantities was tested for the swelling kinetics and the swelling behavior in dependence on the urea concentration of urease-functionalized hydrogels (Figure 7). 

For the swelling kinetics and, therefore, for the response time and for the sensitivity of urea-sensitive hydrogels, no significant differences for the gels with different amounts of enzyme were observed. A doubling as well as a halving of the original enzyme quantity (1 kU/mL) leads to comparable results, especially in the most sensitive range from 0 mmol/L to 20 mmol/L urea. This could also explain the long-term stability of urea-sensitive hydrogels (Section 3.4): Even if small amounts of enzymes diffuse out of the polymer network over time, this has no significant effect on sensitivity or swelling kinetics. 

Furthermore, the swelling behavior of hydrogels was also tested for enzyme amounts of 0.1 kU/mL and 0.25 kU/mL, respectively. However, the results were not reproducible. Some enzyme-functionalized hydrogels swelled strongly in dependence on the urea concentration, whereas other gels showed only low enzyme activities or partially did not swell at all. This indicates a very inhomogeneous distribution of the enzymes in the polymer network. As a result, this could also explain the relatively large measurement uncertainties, especially at higher urea concentrations, in Figure 7.

In summary, a reduction of the enzyme quantity from 1 kU/mL to 0.5 kU/mL is recommended for cost reduction of the sensor. However, a further decrease in the enzyme concentration is not recommended due to the decreasing reproducibility and, therefore, the large measurement uncertainties. Besides the influence on the sensitivity and the response time, the minimization of the enzyme amount could also influence the long-term stability, which needs to be investigated separately before usage in sensing applications. 

### 3.7. Hydrogel-Based Piezoresistive Urea Biosensors

**Figure 8:**
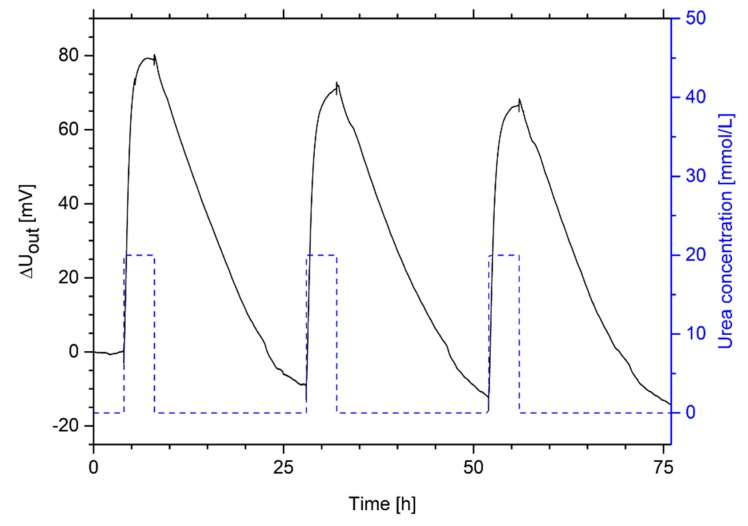
Repeatability of a hydrogel-based piezoresistive urea biosensor—Measurement of the output voltage *ΔU_out_* for cyclic changes of the urea concentration between 0 mmol/L and 20 mmol/L urea.

For sensing applications, the swelling pressure of urea-sensitive hydrogels can be transformed in a measurable output signal via piezoresistive pressure sensors. To show the application potential and for the determination of important sensor properties, hydrogel-based piezoresistive urea biosensors were constructed and measured. The construction corresponds to that described in previous studies [9,10,20]. At first, the repeatability of a hydrogel-based urea sensor was tested via cyclic changes of the urea concentration between 0 mmol/L and 20 mmol/L urea (Figure 8).

As shown in Section 3.4, the measurement of the urea concentration shows good repeatability with respect to the change in output voltage. However, a baseline drift of the signal could be seen as observed also for other kinds of hydrogel-based sensors [20,50,51]. The drift might be caused by the manual sensor set-up and the resulting uncertainties during the sensor preparation [20] and by hysteresis effects often influencing the swelling behavior of hydrogels [28,52,53].

The hydrogel system needs much less time for the swelling process than for the shrinking process. Compared to free-swelling experiments, the response time in sensing applications is much faster. In the free-swelling experiments, the hydrogel system was fully swollen after 24 h and was completely shrunken after two weeks. Due to the smaller size of the hydrogel, the diffusion path is reduced, and the response time was decreased in the sensing application to 4 h for the swelling process and 20 h for the shrinking process. However, commercial applications often require response times in the range of seconds or minutes. 

For example, Shrivastava et al. presented a potentiometric biosensor with a gelatin-entrapped enzyme with a response time of 2 min for the detection of blood urea [54]. Castillo-Ortega et al. developed a conductometric urea biosensor based on poly(aniline)- poly(*n*-butyl methacrylate). The sensor was intended for the detection of urea in serum and had a response time of 10 min [41,55]. Kovács et al. used optical biosensors with entrapped enzymes in a poly(urethane) film for the detection of urea in urine and serum with a response time of ca. 20 s [56]. 

To improve the response time of the tested hydrogel-based piezoresistive urea biosensors, the sensor set-up could be further miniaturized. Furthermore, Franke et al. showed that the response time could be significantly improved by using porous hydrogels [8]. Due to the porous gel structure, the stimulus can diffuse much easier into the gel. However, also the enzyme can diffuse much easier out of the gel. For this purpose, hydrogels with a specific pore size must be produced in order to avoid diffusion of the enzyme. Additionally, the enzymes can be covalently attached to the gel matrix to further advance the long-term stability. 

**Figure 9:**
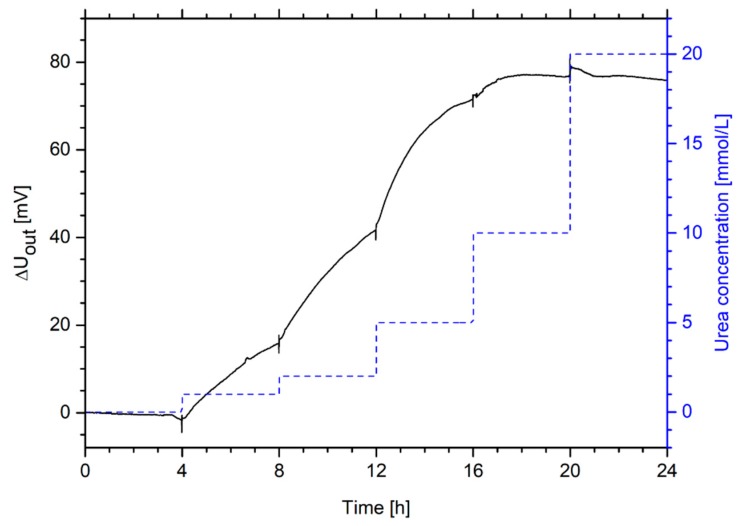
Sensitivity range of a hydrogel-based piezoresistive urea biosensor—Measurement of the output voltage *ΔU_out_* for changes of the urea concentration in a range from 0 mmol/L to 20 mmol/L urea.

To study the sensitivity range of a urea-sensitive hydrogel-based piezoresistive sensor, the urea concentration was increased step-wise up to 20 mmol/L (Figure 9).

The hydrogel sensor is highly sensitive to urea, especially in the range of up to 10 mmol/L urea. The detection limit is smaller than 1 mmol/L urea. For example, the optical urea biosensor by Kovács et al. described above has a linear sensitivity range from 0.07 mmol/L up to 8 mmol/L [56]. Jiménez et al. described a pH-sensitive ion-selective field-effect transistor based on polyacrylamide hydrogels and the entrapped enzyme urease with a sensitivity range from 0.2 mmol/L up to 8 mmol/L [57]. Puig-Lleixà et al. also used this sensor concept by using poly(urethane) for the enzyme immobilization. They could improve the linear sensitivity range from 0.04 mmol/L up to 36 mmol/L [58]. 

## 4. Summary

In this work, we presented an enzyme-functionalized hydrogel system based on poly(acrylic acid-*co*-dimethylaminoethyl methacrylate) hydrogels and the enzyme urease for the detection of urea. Due to the enzymatic reaction, the resulting pH change leads to a swelling of the pH-sensitive hydrogel in dependence on the urea concentration. The hydrogel system showed a high sensitivity in a range from 1 mmol/L up to 20 mmol/L. Due to the repeatable swelling of the hydrogel system, the sensors can be used many times. Compared to other biosensor concepts for the detection of urea, the enzyme-functionalized hydrogels showed an excellent long-term stability over eight weeks without losing any enzymatic activity. Hydrogel-based urea biosensors have a high selectivity to urea compared to similar species. Furthermore, the swelling behavior of enzyme-functionalized hydrogels with different amounts of urease in a range from 0.5 kU/mL to 2 kU/mL was almost similar. Additionally, the working principle of urea-functionalized hydrogel sensors was shown. The urea-sensitive hydrogels have been used in a sensor set-up, where the swelling pressure due to the hydrogel swelling is measured by a piezoresistive pressure sensor. This in-line process-capable hydrogel-based sensor concept could be particularly interesting for future applications due to a high sensitivity and a good long-term stability of the hydrogels as well as a simple and cost-effective sensor set-up.
